# Variational representational similarity analysis

**DOI:** 10.1016/j.neuroimage.2019.06.064

**Published:** 2019-11-01

**Authors:** Karl J. Friston, Jörn Diedrichsen, Emma Holmes, Peter Zeidman

**Affiliations:** a Wellcome Centre for Human Neuroimaging, Institute of Neurology, UCL, WC1N 3AR, UK; b Brain and Mind Institute, Department for Statistical and Actuarial Sciences, Department for Computer Science, University of Western Ontario, Canada

**Keywords:** Representational similarity analysis, RSA, Pattern component modelling, Bayesian, Variational, Multivariate

## Abstract

This technical note describes a variational or Bayesian implementation of representational similarity analysis (RSA) and pattern component modelling (PCM). It considers RSA and PCM as Bayesian model comparison procedures that assess the evidence for stimulus or condition-specific patterns of responses distributed over voxels or channels. On this view, one can use standard variational inference procedures to quantify the contributions of particular patterns to the data, by evaluating second-order parameters or hyperparameters. Crucially, this allows one to use parametric empirical Bayes (PEB) to infer which patterns are consistent among subjects. At the between-subject level, one can then assess the evidence for different (combinations of) hypotheses about condition-specific effects using Bayesian model *comparison*. Alternatively, one can select a single hypothesis that best explains the pattern of responses using Bayesian model *selection*. This note rehearses the technical aspects of within and between-subject RSA using a worked example, as implemented in the Statistical Parametric Mapping (SPM) software. *En route*, we highlight the connection between univariate and multivariate analyses of neuroimaging data and the sorts of analyses that are possible using component modelling and representational similarity analysis.

## Highlights

• We introduce variational RSA, a method for multivariate analysis in neuroimaging.
• This treats RSA a standard covariance component estimation problem.
• An efficient estimation scheme, variational Laplace, is used to estimate parameters.
• Bayesian model comparison is used to optimally test for mixtures of effects.
• We illustrate the approach using simulated and empirical fMRI data.

## 1 Introduction

Functional neuroimaging data usually comprise multivariate timeseries, measured across many voxels or channels. In consequence, the choice of statistical analysis has two aspects: the first concerns the *data* – should each channel be analysed individually, with univariate analyses, or should the data be analysed collectively, using multivariate analyses? Second, should the *hypothesis* be framed in terms of first order responses (e.g., did the treatment change the mean of the data?) or second order effects (e.g., did a treatment change the covariance of the data?). All combinations of these choices call on the same underlying linear model, but with different implementations. This technical note focusses on testing hypotheses about second order effects, using either univariate or multivariate data. In particular, we introduce a framework that implements popular multivariate analysis methods - Representational Similarity Analysis, RSA (Kriegeskorte et al., 2008a) and Pattern Component Modelling, PCM (Diedrichsen et al., 2011, 2018) - using standard variational Bayesian methods. The resulting variational RSA provides statistically efficient tests of competing hypotheses about the patterns that underlie multivariate (and univariate) responses. Additionally, we hope to clarify the formal relationship between covariance component modelling, which forms the basis of PCM and the variational RSA, and classical multivariate statistics (canonical correlation analysis).

### 1.1 Multivariate linear models in neuroimaging

Multivariate analyses are ubiquitous in the neuroimaging literature. These range from applications of classical statistics, such as canonical correlation analysis (aka canonical variate analysis and multivariate analysis of covariance), through to Bayesian procedures inherent in electromagnetic source reconstruction and dynamic causal modelling. In cognitive neuroscience, applications of multivariate PCM and RSA analyses have included the characterisation of motor responses and sequences (Wesselink et al., 2019; Yokoi et al., 2018) and identifying the functional anatomy of stimulus representations in the temporal lobe across species (Connolly et al., 2012; Kriegeskorte et al., 2008a, 2008b).

A comprehensive overview by Diedrichsen and Kriegeskorte (2017) considers multivariate analyses of how experimental conditions elicit distributed responses. These are framed in terms of neuronal representations, where distributed responses are described in terms of *first* and *second*-level parameters. The first-level parameters specify the response of voxels to experimental treatment effects. For example, one can define a parameter for each experimental condition at each voxel – or describe experimental effects in terms of underlying latent features (e.g., stimulus attributes) and define a parameter for each feature at each voxel. The latter approach is especially useful for paradigms that lack discrete experimental conditions – such as parametric designs in which stimuli vary continuously along some feature dimensions. For example, studies using RSA have employed stimuli presented continuously as a movie (Hasson et al., 2004) or while listening to a story (Huth et al., 2016). Conversely, second-level parameters parameterize the distributed responses over a group of voxels or channels (e.g., in a region of interest or searchlight) in terms of the covariance induced by condition specific effects.

The relationship between the parameters and the data they explain is encoded in the general (multivariate) linear model. Using a notation based on (Diedrichsen and Kriegeskorte, 2017), the general linear model (GLM) can be expressed as:

(1) Y=ZU+XB+e

Here, **Y** is a data matrix containing multivariate observations across voxels or channels, in which each row corresponds to one measurement. **Z** is a design matrix specifying the level of experimental factors (e.g., conditions or stimulus features) for each measurement, with a column for each factor. The parameters **U** of the GLM reflect the response at each voxel to each experimental factor, with one row for each factor and a column for each voxel. The corresponding confounds, or nuisance variables – and their first-order parameters – are **X** and **B**, respectively. Finally, the matrix **e** specifies random (within-subject) effects over voxels and observations (i.e., measurement error). We write this matrix in lower case to distinguish it from the expectation operator E.

In this note, an activity *profile* refers to the responses of a particular voxel or channel across different experimental factors (i.e., any given column of the parameter matrix **U**). An activity *pattern* refers to the responses across voxels or channels for a particular experimental factor (e.g. any given row of parameter matrix **U**). Note that we use here “experimental factor” quite generally to refer to a column of the design matrix **Z**. The column could consist of discrete values, e.g. 0 and 1s, encoding the presence of a specific experimental condition or time point in a repeatable natural stimulus, or take on continuous values that encode a stimulus feature.

The sufficient statistics that describe the second order effects of interest are contained within the condition-by-condition covariance matrix **G** = **UU**^T^ (Diedrichsen and Kriegeskorte, 2017). The methods surveyed here – PCM and RSA – both use the linear model of Equation (1), but with different ways of specifying profiles and patterns, which we briefly review before introducing a Bayesian scheme for decomposing **G** into separately estimated components.

### 1.2 PCM and RSA

PCM and RSA differ in how the distributions of activity profiles and activity patterns are specified. In PCM or covariance component estimation, the dimension of matrix **G** is the number of experimental factors. Conceptually, this matrix quantifies the similarities (i.e., the covariances) between the responses elicited by different experimental factors (e.g., stimulus attributes); each value in the matrix represents the covariance between a pair of factors. Conversely, in RSA, the matrix is specified in terms of the *dissimilarity* between the responses elicited by different experimental factors. This matrix is termed a representational dissimilarity matrix (RDM) and each value represents the distance between a pair of factors; for example, the Euclidean or Mahalanobis distance. Mathematically, these two characterisations are roughly equivalent: if a similarity matrix **C** is specified as a correlation matrix and the dissimilarity matrix **D** is based upon the Euclidean distance between patterns, there is a one-to-one mapping between the elements of the representational similarity and dissimilarity matrices^1^; see Equation (4) in (Friston et al., 1996) and Equation (20) in (Diedrichsen and Kriegeskorte, 2017).

(2) $$D_{ij}^{2}=2-2C_{ij}$$

In other words, as the correlation between two stimulus-specific profiles increases, the dissimilarity decreases. If a similarity matrix is specified as a covariance matrix **G**, then a dissimilarity matrix D^*^ can be expressed as;

(3) $$D_{ij}^{*}=G_{ii}+G_{jj}-2G_{ij}$$

Such that **D**^∗^ = **D**^2^ when **G** = **C**. The relative merits of different dissimilarity measures have been considered in the context of RSA (Walther et al., 2016). First, it has been shown that it is beneficial to consider the noise covariance between channels (e.g., voxels) by computing Mahalanobis instead of Euclidian distances. This has been implemented by multivariate noise normalisation (i.e., spatial pre-whitening) of the data, followed by the use of Euclidian distances. By down-weighting spatially correlated noise sources, the distances measures become more reliable. In variational RSA, spatial correlations cannot bias parameter estimates, but they do reduce efficiency, and this is quantified by the spatial degrees of freedom (see the section entitled “Some comments on noise and spatial correlations”). Secondly, with RSA, distance estimates can be (positively) biased by noise, simply because distances cannot be negative. Cross-validation has been suggested as a solution to overcome this problem (Walther et al., 2016). Cross-validated distance estimates are unbiased with an interpretable zero point, thereby furnishing a useful summary statistic. However, in the current treatment, cross-validation is not required because RSA is treated as a covariance component estimation problem (c.f. PCM), where model parameters can be estimated using standard variational methods.

Representational models – i.e., models of the covariance structure induced by treatment effects – are often evaluated by correlating sampled and predicted distances (Nili et al., 2014). While this procedure is intuitive and easy to implement, it is suboptimal (Diedrichsen and Kriegeskorte, 2017), via the Neyman-Pearson lemma. This is because distance estimates are not independent and have a non-uniform variance that is signal-dependent (Diedrichsen et al., 2016). In contrast to covariance matrices, the distributions of RDMs have no analytic forms. For people familiar with multidimensional scaling, the difference between PCM and RSA echoes the difference between metric and non-metric multidimensional scaling, where the former can be reduced to principle coordinates or components analysis, while the latter cannot. For further discussion please see (Friston et al., 1996), which addresses multidimensional scaling in the context of neuroimaging.

In summary, PCM and RSA are predicated on the same underlying generative (standard linear mixed) model and both have the same objective; namely, inferring the contribution of various components to an observed response. This means they differ only in their implementation. As evident from Equations (2), (3), one can always derive RDMs from second-order matrices or covariance components (Diedrichsen and Kriegeskorte, 2017). Strategically, this means that we can model the components of the covariance matrix directly and use variational approaches to make inferences.

### 1.3 Variational RSA

Our objective is to decompose the matrix **G** into a mixture of covariance components, where each component embodies a hypothesis, and to infer the hyperparameters controlling the contribution of each component (Dempster et al., 1981; Harville, 1974). The particular scheme described below is a standard approximate Bayesian inference scheme called Variational Laplace (Friston et al., 2007), which assumes that the posteriors over unknown parameters and hyperparameters are Gaussian. This is exactly the same scheme used in electromagnetic source reconstruction to solve the implicit spatial covariance component problem (Friston et al., 2008b) and in multivariate Bayes (MVB) to decode brain images (Friston et al., 2008a). In applications to source reconstruction, the covariance components correspond to patterns of responses induced by activity in each electromagnetic source in the forward model. In this paper, Variational Laplace (VL) is used in the setting of pattern component modelling and representational similarity analysis (Diedrichsen and Kriegeskorte, 2017). See also (Cai et al., 2016, 2019), who introduce a Bayesian formulation of representational similarity, to reduce estimation bias by modelling error covariance.

The key advantages of variational RSA include:

• Optimal efficiency and inference, in virtue of using marginal likelihoods (i.e., implicit Bayes factors).
• Robust between-subject analyses using parametric empirical Bayes (i.e., hierarchical Bayesian modelling).
• Bayesian credible intervals on the contribution of hypothesis matrices (e.g. covariance components) at the within and between-subject levels.
• Flexible Bayesian model comparison and selection at the within and between-subject levels.
• A formal and analytic connection to standard characterisations of first-order statistics (e.g., canonical variate analysis).
• An optimal and straightforward way of dealing with spatial correlations (which eschews spatial pre-whitening).
• Graceful handling of (e.g., correlated) hypothesis matrices that can have arbitrary correlation structures.
• Computational expediency, in virtue of using variational Bayes (as opposed to sampling or cross-validation).

Typically, in RSA, hypotheses are specified in terms of RDMs. Similarly, variational RSA allows one to formulate hypothesis about distributed response components in terms of similarity or covariance matrices that we will refer to as *hypothesis matrices*. Using standard variational procedures, one can then evaluate the contribution of each of component to a multivariate response (e.g., across voxels or channels). These contributions (i.e., hyperparameters) can then be analysed at the between-subject level, allowing for random effects on the contributions of various components. Finally, one can perform Bayesian model *comparison* to assess the evidence for different components or Bayesian model *selection* to select the best component (i.e., to categorise the pattern of responses in terms of one of several possible components).

Crucially, the hypothesis matrices—and implicit components—can come in two flavours. They can either describe a single feature or a mixture of multiple features (where a feature is, for example, an experimental condition, a contrast of conditions, or a continuous variable with a value for each condition or stimulus). Mathematically, this difference corresponds to the rank of the hypothesis matrix, which can be equal to or greater than one. This means one can decompose any hypothesis matrix into its underlying principal components (i.e., eigenvectors) or specify a component as a particular mixture of orthogonal patterns. The scheme described below can accommodate either – and we will illustrate the differences using worked examples. If one chooses to decompose a hypothesis matrix into underlying orthogonal features, a separate hyperparameter is associated with each feature. Testing for a single feature reduces to a test for the corresponding contrast of experimental effects (i.e., a rank one hypothesis matrix). We will return to this special case in Section 2.4: Contrasts and hypothesis matrices.

In what follows, we briefly describe the technical steps entailed by variational RSA and provide two worked examples. The first uses simulated data with the kind of experimental design that is typically employed with RSA analyses. The second uses empirical data to illustrate variational RSA analysis with a ‘searchlight’ approach over the brain. The data and associated analysis scripts are available as part of the Statistical Parametric Mapping (SPM) software.

## 2 Theory

### 2.1 The generative model

We start with the multivariate GLM in Equation (1). The only distributional assumption is that the errors $e\in {\mathbb{R}}^{m\times p}$ are independently and identically distributed over *m* measurements within a voxel but can show (spatial) covariance $V\in {\mathbb{R}}^{p\times p}$ over *p* voxels:

(4) $$\begin{array}{lll} vec\left(e\right) & ∼ & N\left(0,V⊗I_{m}\right)\\ & \Rightarrow & ee^{T}∼W_{m}\left(I_{m},v_{e}\right)\\ & \Rightarrow & E\left[ee^{T}\right]=v_{e}\cdot I_{m}\\ v_{e} & = & \frac{tr\left(V\right)tr\left(V\right)}{tr\left(VV\right)} \end{array}$$

Where *W*_*m*_ is the Wishart distribution of dimension *m*. This means that the expected second-order matrix of errors $ee^{T}\in {\mathbb{R}}^{m\times m}$ over *features* or time is the identity matrix scaled by the *spatial* degrees of freedom *v*_*e*_ due to spatial correlations (Seber, 1977; Worsley and Friston, 1995). Spatial degrees of freedom play exactly the same role as the effective degrees of freedom of serially correlated fMRI timeseries.

In this form, the GLM is parameterised in terms of first-order parameters and could be inverted in a number of ways. Classically, one would use canonical variate analysis (CVA); aka canonical correlation analysis, multivariate analysis of variance (MANOVA) or, more generally, a multivariate linear model. However, for the purpose of inferring distributed responses or profiles, we are not interested in the first-order parameters **U** *per se*. Rather, we are interested in the second-moment matrix **G** = **UU**^*T*^, which summarises the response profile over experimental factors. In other words, we are not interested in the *pattern* of spatial responses to – or encoding of – experimental factors, we are only interested in relationship between these patterns in terms of their profiles over features or experimental factors.

This means we need to make inferences about the second-order matrix **G**. To do this, we multiply the GLM by the generalised inverse of the design matrix $Z^{-}$ and then remove confounds^2^ with an idempotent residual forming matrix **R** = **R**^*T*^:

(5) $$\begin{array}{lll} RZ^{-}Y & ≜ & \hat{U}=RU+RZ^{-}e\\ R & = & I-\left(Z^{-}X\right){\left(Z^{-}X\right)}^{-} \end{array}$$

We can now create a second-order form by multiplying both sides of the equation by their transposed versions and taking an expectation. This allows us to express second-order data features **S** as a mixture of covariance components due to responses and measurement error (noting that the response profiles are, in expectation, not correlated with measurement error):

(6) $$\begin{array}{lll} S & ≜ & \hat{U}{\hat{U}}^{T}\\ & = & RUU^{T}R+E\left[RZ^{-}ee^{T}Z^{-T}R\right]\\ & = & RGR+v_{e}RC_{e}R\\ G & = & UU^{T}\\ C_{e} & = & Z^{-}Z^{-T} \end{array}$$

Note that the second-order data features are also the outer products of the maximum likelihood estimates of the first-order parameters. In this form, the first-order parameters **U** have been replaced by second-order matrix **G** – that can be regarded as the responses induced by different conditions or features over voxels. The error term has been parameterised by the nonnegative (scale) parameter *v*_*e*_ that corresponds to the spatial degrees of freedom.

We can now express the second-order parameters in terms of a mixture of hypothesis matrices or covariance components **C**. Each of these components can be thought of as an experimental factor or feature selective component that constitutes the measured responses:

(7) $$\begin{array}{lll} G & = & UU^{T}=v_{1}C_{1}+v_{2}C_{2}+\ldots \\ v_{i} & = & \exp\left(\lambda_{i}\right)\\ p\left(\lambda \right) & = & N\left(\eta ,\Sigma \right) \end{array}$$

The contribution of each component is controlled by a (nonnegative) scale parameter *v* that has a lognormal distribution to ensure positivity. This constraint is required to ensure that the weighted sum of the components is a proper covariance matrix (i.e. positive semi-definite). Notice that expressing **G** as the sum of covariance components does not assume that each component is independent; for example, the tuning of a region for shape may depend on its tuning for colour, via the dependencies among the hyperparameters $\lambda_{i}=\ln v_{i}$. These dependencies are encoded in the estimated covariance matrix.

The crucial step in variational RSA is the introduction of a prior on these hyperparameters (sometimes known as hyperpriors). This allows hypotheses to be tested in terms of particular covariance components using Bayesian model comparison. In other words, we can evaluate the evidence for models with and without particular combinations of covariance components. The Bayesian methods used here mean that these comparisons consider the full covariance among the hyperparameters. Furthermore, it allows us to apply parametric empirical Bayes and deal with random effects at the between subject level in a proper fashion (see below).

Equation (7) shows that the prior over the scale parameters *v*_*i*_ is log-normal (or equivalently, a normal prior on the log scale parameters *λ*_*i*_). In the examples which follow, the prior expectation of *v*_*i*_ is set to a value close to zero, thereby realising the null hypothesis that the corresponding covariance component is negligible. This is implemented by setting the prior expectation of the log scaling parameter to η=−16, which means the prior expectation *E*[*p*(*v*_*i*_)] =* *exp(−16) = 1.12*e*−7 is nearly zero. Hyperpriors like this are key in variational RSA, because they enable Bayesian model comparison and parametric empirical Bayes. In general applications, hyperpriors of this sort are usually uninformative. Although not pursued here, there is an interesting opportunity to restrict various covariance components according to prior beliefs—or indeed implement a regularised or constrained solution, for which the degree of regularisation could itself be optimised using Variational Laplace.

Notice that we are describing the hypothesis matrices as covariance components. This presupposes that the rows of the data matrix have been mean centred. In other words, we are assuming that people are interested in the feature or functional selectivity of responses in terms of a deviation from the average response induced by a particular condition or experimental factor over voxels. This converts the second-order matrices into covariance matrices. There may or may not be good motivations for retaining the spatial mean in the second-order response matrix: see (Diedrichsen and Kriegeskorte, 2017) for discussion. Here, we will assume that people would typically characterise the mean response (with standard univariate analyses) and use (orthogonal) fluctuations about the mean (with RSA), to disambiguate regionally specific responses from profiles with no spatial specificity. This assumption sidesteps the potential issue of nonlinear responses (e.g., when responses are proportional to mean activity), which generally calls for nonlinear transforms of the data or nonlinear models.

Because the hyperparameters have, *a priori*, a Gaussian distribution, we can now use Variational Laplace to estimate the (Gaussian) posterior over each hyperparameter, under appropriate (uninformative) priors. In what follows, we will assume *a priori* that the contribution has a small prior expectation but a large variance, with an expectation of η=−16 and a prior variance of Σ=128. Notice that this generative model entails prior beliefs about the hyperparameters or contribution of each component. This is standard practice in most applications of this variational scheme and differentiates it from *ad hoc* schemes^3^ like restricted maximum likelihood (Harville, 1974).

### 2.2 Variational Laplace

In brief, variational approaches rest on minimising a quantity called the Feynman variational bound, or negative free energy (Feynman, 1972). Variational free energy represents a bound on the log-evidence ln *p*(**Y**) also known as an evidence lower bound (ELBO) in machine learning. Variational methods are well established in the approximation of densities in statistical physics; e.g., Weissbach et al. (2002). The variational framework was introduced into machine learning though ensemble learning (Hinton and Van Camp, 1993; MacKay, 1995a, b). Later, schemes like expectation maximisation (EM) were considered in the light of variational Bayes (VB) (Beal, 2003; Bishop, 1998; Neal and Hinton, 1998), which proved useful in a variety of domains, particularly with graphical models (Jordan et al., 1999). A generic variational scheme, commonly used in neuroimaging, is Variational Laplace (VL), which involves optimising the sufficient statistics of a Gaussian posterior with respect to the variational free energy (Friston et al., 2007). This scheme is generic because it does not require the use of conjugate priors and can be applied, in principle, to any generative model. In short, when variational free energy is maximised, the (approximate) posterior converges to the true posterior while, at the same time, the free energy becomes the log model evidence. This will be important later when we use free energy for Bayesian model comparison. Mathematically, this can be summarised as follows:

(8) $$\begin{array}{lll} p\left(\lambda \right) & = & N\left(\eta ,\Sigma \right)\\ q\left(\lambda \right) & = & N\left(\mu ,\Omega \right)\\ q^{*} & = & \mathrm{argmax}F\left[q\left(\lambda \right),p\left(\lambda \right),Y\right]\\ F & = & E_{q}\left[\ln\;p\left(Y|\lambda \right)p\left(\lambda \right)-\ln\;q\left(\lambda \right)\right]\\ q^{*} & \approx & p\left(\lambda |Y\right)\\ F\left[q^{*},p\left(\lambda \right),Y\right] & \approx & \ln\;P\left(Y:p\left(\lambda \right)\right) \end{array}$$

The notation P(Y:p(λ)) means the probability of observing **Y** under some prior assumptions p(λ) about the hyperparameters. Here, these priors are Gaussian shrinkage priors, which make minimal assumptions – simply ensuring that (in the absence of evidence) each covariance component's contribution will shrink to zero. The posterior density over the hyperparameters is approximated by the Gaussian density q(λ)=N(μ,Ω). In our case, the variational free energy is given by Equation (18) in (Friston et al., 2008a), where (ignoring constants):

(9) $$\begin{array}{lll} F & = & -\frac{1}{2}\left(tr\left({\hat{S}}^{-1}S\right)+\upsilon \;\ln|\hat{S}|+\ln|\Sigma^{-1}\Omega |-{\left(\mu -\eta \right)}^{T}\Sigma^{-1}\left(\mu -\eta \right)\right)\\ \hat{S} & = & {\overset{⌢}{v}}_{1}C_{1}+{\overset{⌢}{v}}_{2}C_{2}+\ldots {\overset{⌢}{v}}_{e}C_{e}\\ {\overset{⌢}{v}}_{i} & = & \exp\left(\mu_{i}\right) \end{array}$$

Here, $\hat{S}$ can be regarded as a prediction of the second-order data matrix based upon the posterior expectations of the hyperparameters. This can be compared with the simpler (restricted maximum likelihood) objective functions in Equation (26) in (Friston et al., 2007) and Equation (15) in (Diedrichsen and Kriegeskorte, 2017), which do not consider hyperpriors.

The free energy depends upon the number of covariance components (*n*) and the effective number of voxels (*υ*) in the second-order data matrix. These can be computed from the spatial residuals follows:

(10) $$\begin{array}{lll} \upsilon & = & \frac{tr\left(ϒ\right)tr\left(ϒ\right)}{tr\left(ϒϒ\right)}\\ ϒ & = & r^{T}r\\ r & = & Y-\left[Z,X\right]{\left[Z,X\right]}^{-}Y \end{array}$$

This quantity scores the effective spatial degrees of freedom and accounts for spatial correlations. In other words, if the errors (or more precisely the residuals) at each voxel were completely uncorrelated, the above expression shows that the effective degrees of freedom are equal to the number of voxels (because ϒ would be an identity matrix). Conversely, in the setting of complete correlations, the effective degrees of freedom reduce to one (i.e., functional selectivity is completely expressed in terms of the mean over voxels).

Variational schemes may be contrasted against sampling methods (e.g., MCMC), which provide a gold standard for evaluating posterior distributions (Blei et al., 2017). However, sampling methods have well-known difficulties in evaluating model evidence, which is required for model comparison and selection. Furthermore, variational methods are computationally more efficient – and are generally preferred when dealing with well-behaved models. As illustrated in the empirical example that follows, performing a ‘searchlight’ RSA requires a model inversion for every voxel. The use of VL enabled the estimation of 29,319 models (covering all grey matter voxels) in a few minutes, using a standard desktop computer without parallelisation.

With only a single subject and session, we could proceed directly to Bayesian model comparison and make inferences about the contribution of any particular component. For example, we could compare the log-evidence (i.e., variational free energy) between the full RSA model and a reduced model in which one hyperparameter is fixed to zero using precise hyperpriors to remove the influence of the corresponding experimental effect. However, neuroimaging experiments typically have multiple subjects or sessions – and one generally wants to evaluate the contributions of different components that are conserved over subjects. This suggests the use of a hierarchical model of these contributions (i.e., hyperparameters), which we now turn to.

### 2.3 Parametric empirical Bayes

Analyses over subjects or sessions are simply implemented using a second GLM at the between-subject level, with a procedure known as parametric empirical Bayes (Efron and Morris, 1973; Kass and Steffey, 1989). This equips the generative model with an extra (between-subject) level and accommodates random effects on the hyperparameters over subjects—and uncertainty about subject-specific estimates—to furnish a Bayes-optimal posterior over the average hyperparameters.

Formally, the second level model generates subject-specific contributions from *n* components $\lambda \in {\mathbb{R}}^{s\times n}$ for *s* subjects from a between-subject design matrix $D\in {\mathbb{R}}^{s\times r}$, with *r* regressors. The first regressor is a column of ones that captures the average effects across subjects, and subsequent regressors capture remaining subject-specific effects of interest (e.g., age):

(11) $$\lambda =D\lambda^{\left(2\right)}+r$$

Here, $\lambda^{\left(2\right)}\in {\mathbb{R}}^{r\times n}$ are second-level or between-subject effects, which are estimated from the data, and **r** is additive between-subject variability (i.e., random effects). Adding this between-subject level places empirical priors on the contribution or hyperparameter estimates from all subjects. Expressed in terms of minimising variational free energy, we have:

(12) $$\begin{array}{lll} p\left(\lambda^{\left(2\right)}\right) & = & N\left(\eta ,\Sigma \right)\\ q\left(\lambda^{\left(2\right)}\right) & = & N\left(\mu ,\Omega \right)\\ q^{*} & = & \mathrm{argmax}F\left[q\left(\lambda^{\left(2\right)}\right),p\left(\lambda \right),p\left(\lambda^{\left(2\right)}\right),Y_{1},\ldots ,Y_{S}\right]\\ q^{*} & \approx & p\left(\lambda^{\left(2\right)}|Y_{1},\ldots ,Y_{S}\right)\\ F\left[q^{*},p\left(\lambda \right),p\left(\lambda^{\left(2\right)}\right),Y_{1},\ldots ,Y_{S}\right] & \approx & \ln\;P\left(Y_{1},\ldots ,Y_{S}:p\left(\lambda \right),p\left(\lambda^{\left(2\right)}\right)\right) \end{array}$$

Here, bold variables represent the corresponding subject-specific variables at the between-subject level and **Y**_1_,…,**Y**_*S*_ denotes the data from all subjects. Practically, the optimisation of the posterior over group effects can be implemented efficiently using the within-subject posteriors (and priors) as described in (Friston et al., 2016). In the typical PEB approach, constraints on individual subjects are applied by re-estimating each subject's model using the group-level posteriors as empirical priors. The approach used here, called *Bayesian Model Reduction*, analytically computes the posteriors one would expect for each subject, given empirical priors from the group. This means it is only necessary to estimate the contribution for each subject once and then estimate the posterior density over group effects in a single step. To demonstrate the practical aspects of the scheme, we will introduce a simulated dataset that will be used to illustrate Bayesian model comparison. However, first, it will be useful to establish the relationship between estimates of first and second order parameters of any given GLM.

### 2.4 Contrasts and hypothesis matrices

Finally, we consider the relationship between the hypothesis matrices, contrasts, and canonical vectors used with the GLM in Equation (1). There is a straightforward relationship between these characterisations of condition-specific responses. This relationship can be seen clearly if we decompose the second-order matrix **G** into its principal components or orthogonal patterns (using, for example, singular value decomposition)

$$G=cvc^{T}:c^{T}c=I$$

Here, *v* is a diagonal matrix of eigenvalues and *c* is an orthonormal matrix of eigenvectors (or singular vectors). This means we can decompose any given response into series of single rank hypothesis matrices; each defined by a vector over experimental levels – that defines the profile and component in question. From Equation (7) we have:

(13) $$\begin{array}{lll} G & = & UU^{T}\\ & = & v_{1}C_{1}+v_{2}C_{2}+\ldots \\ & = & c_{1}v_{1}c_{1}^{T}+c_{2}v_{2}c_{2}^{T}+\ldots \\ & \Rightarrow & \\ \sqrt{v_{i}} & = & c_{i}^{T}U \end{array}$$

These equalities mean that the square root of the contribution is just the *contrast of first-order parameters*. In other words, rank-one hypotheses play exactly the same role as a contrast of parameter estimates used to specify tests for particular patterns in classical analyses; such as canonical variates analysis. In the absence of any specified contrast, canonical variates analysis will identify the ‘best’ patterns that are expressed to the greatest extent, relative to measurement noise. In this context, *c*_*i*_ and *v*_*i*_ are referred to as *canonical vectors* and *canonical values,* respectively. In other words, in the absence of a specific hypothesis about pattern components, the best hypothesis is a mixture of canonical vectors or patterns, weighted by their canonical values or contributions. See (Friston et al., 1995) for a detailed discussion in the context of neuroimaging.

On this view, the hypothesis matrix defines a subspace of the design matrix that we want to make an inference about. The difference between a hypothesis matrix with a rank of one and a rank greater than one is analogous to the difference between a *t*-contrast and *F*-contrast in classical inference. In other words, both specify subspaces of the design (i.e., experimental conditions or stimulus attributes), where this subspace can be a single pattern or can span a mixture of patterns. When testing for contrasts of first-order parameters with MANOVA or canonical variate analysis, a rank one hypothesis matrix specifies a *t*-contrast and the resulting test is known as a Hotelling's T-squared (Hotelling, 1931). Otherwise, the classical tests for multivariate responses are based on Wilk's Lambda (Friston et al., 1995).

In some situations, the hypothesis matrix may be of full rank. For example, it could be an empirical covariance matrix taken from another region, or indeed, another experiment or species. When the rank of the hypothesis matrix exceeds one, there is an opportunity to specify a single contrast or hypothesis that has a particular mixture of orthogonal patterns—or specify each orthogonal pattern separately as a rank one hypothesis. In other words, one can decompose any hypothesis matrix into a series of orthogonal rank one hypotheses using singular value decomposition:

(14) $$\begin{array}{lll} c & = & \left[c_{1},\ldots ,c_{N}\right]=SVD\left(C\right)\\ C_{i} & = & c_{i}c_{i}^{T} \end{array}$$

**Fig. 1:**
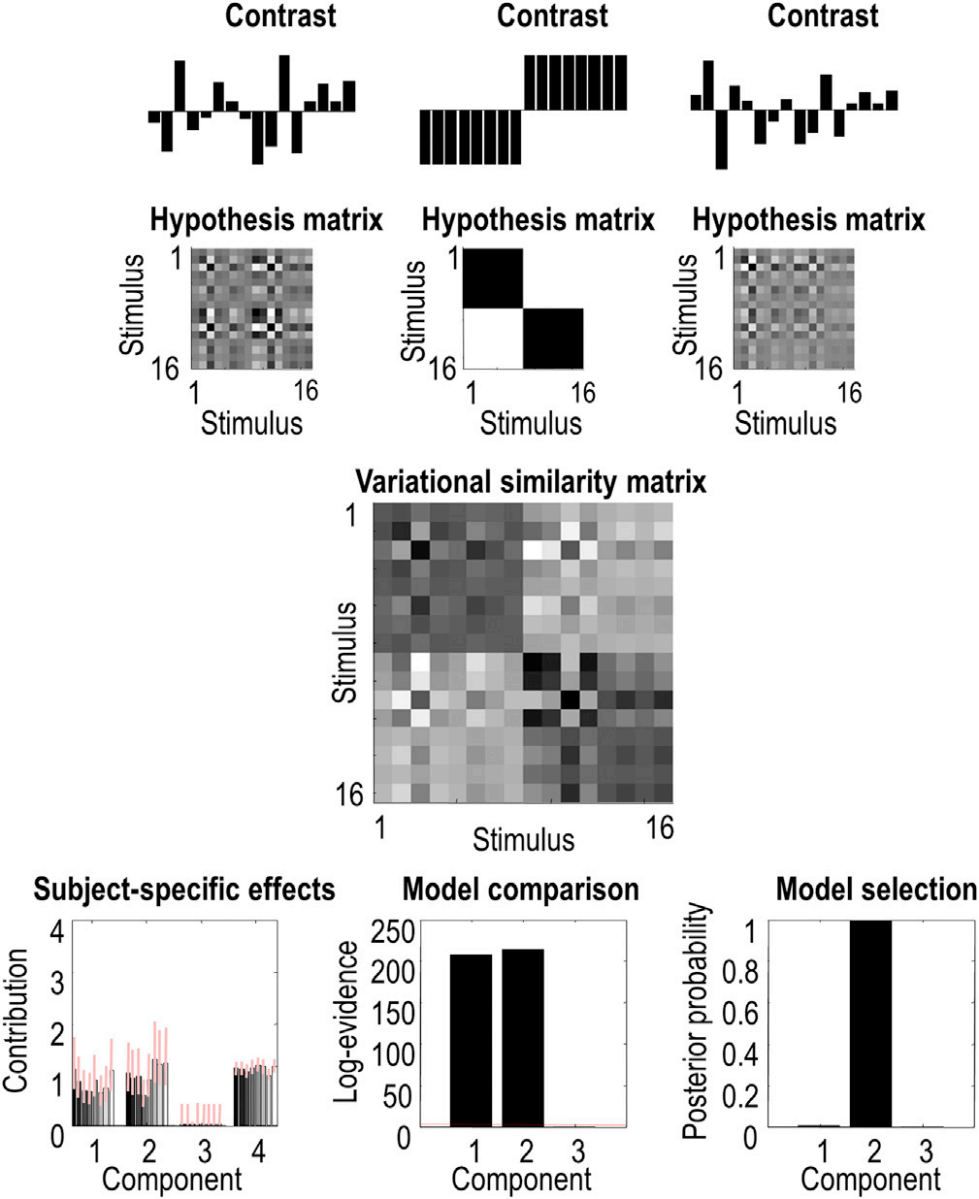
This figure reports the simulated experimental design (upper panel) and the results of a Variational RSA (middle and bottom panels), illustrating the steps entailed by a Variational RSA of multisubject data. This output is in the format used by the SPM software (please see software note). In this example, data were generated using two main effects but no interaction. **Upper panel**: The bar charts show contrast vectors corresponding to the two main effects and their interaction respectively, with 1 bar per stimulus. The interaction is just the product of the two main effects. The second row illustrates these patterns as *hypothesis matrices* or *covariance components****C***_1_, …, ***C***_3_, by taking the outer products of the corresponding contrast vector. **Middle panel:** Estimated variational similarity matrix $\hat{S}$, which reflects a mixture of the two main effects (of the parametric and categorical factors; components 1–2) used to generate the data, with a negligible contribution from the interaction (component 3). **Lower left panel**: Posterior density over each subject's contribution (i.e., exponentiated hyperparameter) to the simulated data. Each bar represents the posterior expectation of a hyperparameter from a single subject, grouped according to the two main effects (components 1–2), the interaction (component 3) and the contribution of measurement noise (component 4). The 90% Bayesian credible (i.e., confidence) intervals are shown as pink error bars. **Lower middle panel**: Bayesian model comparison based upon the log-evidence for each component at the second (between-subject) level. The bars quantify the difference in evidence between models that do and do not contain each component, as evaluated using Bayesian model reduction. A relative log evidence of 3 (red dotted horizontal line) corresponds to a Bayes factor of about exp(3) ≈ 20 to 1. This difference indicates strong evidence that a component contributes to the observed data. **Lower right panel**: This graph shows the posterior probability of models that contain one (and only one) of the three components in the upper row. This indicates what would happen if we assumed that the simulated region could only express one of the three components. Here, this would be slightly disingenuous, because we deliberately simulated the expression of two patterns in the data.

Using rank one hypothesis matrices, **C**_*i*_ corresponds to a test for main effects and interactions in the usual way. In this instance, the hypothesis matrices can provide a useful visualisation of the corresponding treatment effect one is testing for (see Fig. 1 for example). However, when using hypothesis matrices whose rank is greater than one, the particular mixture of experimental effects may or may not be easily related to designed experimental effects. In this setting, it is assumed that this particular mixture has some meaning or validity that underwrites subsequent Bayesian model comparison. In short, to say that this pattern is prevalent in this region is only interesting if the pattern encoded by the hypothesis matrix has a useful interpretation (e.g., the mixture of patterns seen in another part of the brain, or perhaps in another species). See (Diedrichsen and Kriegeskorte, 2017).

## 3 Simulated example

To illustrate the various steps entailed by a variational RSA of multisubject data, we simulated data from 8 subjects, viewing 16 stimuli. The experimental design had one parametric factor (for example, the valence, brightness or loudness of a stimulus) and one categorical factor (for example, attended versus ignored or coloured versus moving). Thus, our experiment had two main effects and one interaction. In this example, the response contained both main effects in equal measure, but no interaction.

The upper panel of Fig. 1 shows the main effects and interaction as contrast vectors (bar charts) with one value per stimulus. It also illustrates the same effects as hypothesis matrices, which are calculated by taking the outer products of the contrast vectors. These three hypothesis matrices or covariance components have rank one. Note that through singular value decomposition (SVD), the hypothesis matrices can be converted back into the contrast vectors displayed in the bar charts (in the upper panel). We used this experimental design to generate simulated data (using 24 presentations of each of the 16 stimuli) for eight subjects. Each subject's observation noise was randomly sampled from the same multivariate normal density, with standard deviation set to a half of the simulated main effects.

### 3.1 Model inversion

We inverted the general linear model (GLM) for each subject. This model was encoded in a design matrix, where the associated confounds comprised a column of ones (to model a constant response over observations). The resulting contributions of the three components (i.e., hyperparameters), as well as the hyperparameter controlling the precision of the observation noise, were estimated using Variational Laplace (VL) for each subject.

The subject-specific posteriors over hyperparameters were then analysed using Parametric Empirical Bayes (PEB), to produce a posterior estimate of the group average at the between-subject level. The estimated mixture of the two main effects (components corresponding to the parametric and categorical factors) are shown in the middle panel of Fig. 1. This mixture corresponds to the estimate of matrix **G** in Equation (7). Additionally, this procedure updates the hyperparameters of each subject, by using the group-level posteriors as empirical priors. The lower left panel of Fig. 1 shows the ensuing posterior density for each subject. The first three groups of bars correspond to the three experimental effects (i.e., exp(λ) from Equation (11)) and the fourth group corresponds to the precision of the observation noise. Note that the contribution of the two main effects has been correctly identified as present (non-zero), whereas the interaction has been properly estimated to be (nearly) absent.

### 3.2 Bayesian model comparison and selection

We now have a full posterior over the conserved or average hyperparameters – and are in a position to make inferences about contributions of each hyperparameter (i.e., component) using Bayesian model comparison. We can do this by comparing the log-evidence (i.e., free energy) between our group-level model and the same model when one hyperparameter is ‘shrunk’ towards zero with very precise hyperpriors; essentially removing its contribution. Because we are dealing with log scale hyperparameters, this corresponds to placing a precise shrinkage prior on the prior expectation η=−16. In other words, we replace uninformative priors (variance Σ=128) with precise priors (variance Σ=1/128), suppressing the contribution of particular components to the model. To score the evidence for the contribution of each component, the ensuing change in log-evidence can be evaluated analytically (under the Laplace assumption) using Bayesian model reduction (BMR) (Friston et al., 2016); see the lower middle panel in Fig. 1.

Alternatively, one could assume, *a priori*, that the brain region in question can only express one of a specified number of components, as is frequently assumed in computational neuroimaging studies. In other words, each of the hypothesis matrices represents a mutually exclusive or competing explanation for observed responses. This would correspond to Bayesian model selection over competing and exclusive hypotheses – and can be implemented using a softmax function of the appropriate log evidences; namely, the log-evidence for models with one and only one component. The use of the softmax function or normalised exponential effectively applies a sum to one constraint over single component models; thereby treating them as competing explanations for the same data. See the lower right panel in Fig. 1 for an example of applying this extra (exclusion) prior.

Notice an important but subtle distinction between the two sorts of inference. In one case, we are saying that a region can contain a mixture of different components—and we are inferring the presence of responses associated with each component separately using Bayesian model *comparison*. Whereas, Bayesian model *selection* among components adopts the alternative view that a region must be responding according to one (and only one) of the hypotheses. Under Bayesian model selection, we use the log-evidence to select the most likely model that best describes the data. Both types of inference are easily accessed using the current scheme. Due to the speed with which models can be compared using Bayesian model reduction, it is possible to compare thousands of reduced models and select the optimal combination of hyperparameters for the data in a matter of milliseconds (Friston et al., 2016).

### 3.3 Some comments on noise and spatial correlations

**Fig. 2:**
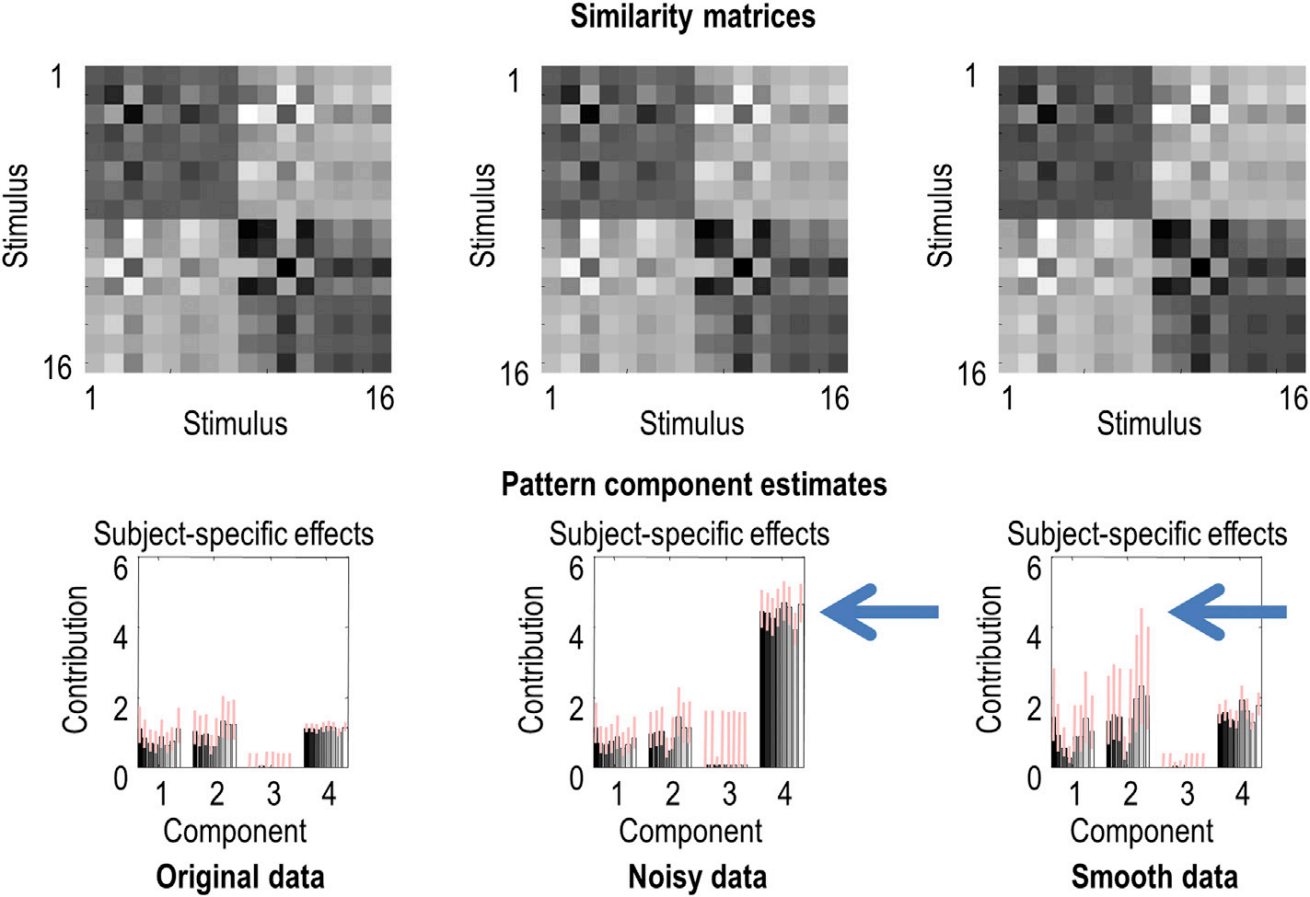
This figure replicates results of the previous figure using higher levels of noise and spatial correlations. The **top row** reports the similarity matrices based upon the group level parametric empirical Bayes estimators for three analyses, while the **bottom row** shows the underlying subject-specific effects in terms of posterior means and Bayesian credible intervals (bars and pink lines), as in the previous figure. The **left column** reproduces the results reported in Fig. 1. The **middle column** shows the corresponding results using exactly the same data but after scaling the measurement noise by a factor of two. This means the contribution or variance attributed to the noise component increases, on average, from 1 to 4. Note that the covariance components associated with condition-specific effects are virtually unaffected. The **right** column uses the original level of measurement noise (with a standard deviation of one) but increases the smoothness of the data from a standard deviation of an eighth of a voxel to 1 voxel. This eightfold increase in spatial correlations increases the variability of the covariance parameter estimates and, more importantly, the Bayesian credible or confidence intervals. In other words, spatial dependencies reduce the degrees of freedom inherent in the data, decreasing the efficiency of the estimates.

An interesting aspect of covariance or pattern component analysis is that they are not confounded by high levels of measurement noise. In other words, the estimates of the hyperparameters do not change systematically with higher levels of measurement error. This may seem counterintuitive; however, the effect of measurement noise on estimators of first and second-order parameters is quite different. Although noise can bias standard representational distance estimates, it has little effect on estimates of the contribution of each covariance component. This is because the noise is just another covariance component that has a particular (spherical) form. This is illustrated in Fig. 2 where we doubled the level of measurement noise, thereby increasing its variance or contribution from 1 to 4. This is reflected fairly accurately in the results of the variational RSA, with almost no effect on the posterior expectations and covariances of the second order parameters (compare left and middle rows of Fig. 2).^4^

In contrast, spatial correlations or smoothness can affect efficiency, via the effective degrees of freedom in Equation (10). In other words, although spatial correlations cannot bias the estimates, they can directly reduce the efficiency or increase the uncertainty about those estimates (Cai et al., 2016). This follows because the form of the spatial correlations cannot, on average, influence the form of the covariance over time or features. This is illustrated in the right column of Fig. 2. Here, instead of increasing the level of measurement noise, we increased the degree of smoothness in the data by a factor of eight. The key consequence of this is an increase in the variability of the expected contributions and, more importantly, an increase in their Bayesian confidence intervals. In short, the smoothness or spatial dependencies in the data effectively determine the degrees of freedom available for precise inference about covariance components. This is the same phenomenon that underlies random field theory corrections for multiple comparisons in topological inference (i.e., statistical parametric mapping). In this setting, the effective voxel size or resolution element is called a RESEL (Friston et al., 1994; Worsley et al., 1996).

With variational RSA, treatment effects (i.e., condition-specific responses) and random effects (i.e., noise) are treated on an equal footing: they are both just covariance components. In the analyses presented in this paper, these have been assumed to be identically and independently distributed. However, it is easy to estimate condition specific error components by replacing the single independently and identically distributed (IID) noise component with a series of components whose leading diagonal elements model condition-specific noise variances (or indeed, serial correlations when applying RSA directly to timeseries). Furthermore, one can use Bayesian model comparison to assess the evidence for IID assumptions, relative to any other (non-spherical) noise structure. This aspect of covariance component modelling is used routinely to deal with non-sphericity (i.e., departure from identity and independence assumptions) in repeated measures designs or when dealing with serially correlated data (Friston et al., 2007).

This section has illustrated the intimate relationship between classical analyses of first-order responses and characterisations of single-rank second order hypotheses. In the next section, we apply variational RSA to empirical data to illustrate how one can test for functionally selective brain responses that are ‘similar’ to some seed or target region, with a functional specialisation that spans more than one stimulus feature or attribute.

## 4 Empirical example

This section illustrates variational RSA in the context of an fMRI experiment investigating the perception of visual motion (Buchel and Friston, 1997). This is a well characterised dataset that has been used to demonstrate many functional analysis methods in SPM. The fMRI data were acquired from a single subject who viewed dots displayed on a computer screen in the MRI scanner. Following a block design, the dots were either in motion or stationary, and the subject was asked to either pay attention to the speed of the moving dots or to watch them passively.

### 4.1 Data and design

We focussed our first analysis of these data on the motion-sensitive visual region V5. To select relevant timeseries, we first performed a standard mass-univariate General Linear Model (GLM) analysis, with regressors encoding the experimental conditions: motion with attention, motion without attention, static dots, and a constant term. We then computed a statistical parametric map for the main effect of motion (contrast vector: [1 1–2 0]), thresholded at p < 0.05 (family-wise error corrected). We identified the closest peak to left V5 (MNI -45, −69, 0), based on the V5 probability map from the Neurosynth analysis tool (Yarkoni et al., 2011), and extracted timeseries from the 19 supra-threshold voxels that were within 8 mm of the peak. Following standard procedures in SPM, these timeseries were high-pass filtered, whitened and mean-corrected. We additionally mean-corrected measurements over voxels; i.e., each row of the data matrix $Y\in {\mathbb{R}}^{m\times p}$ with *m* = 360 measurements and *p* = 19 voxels.

For the variational RSA analysis, the design matrix $Z\in {\mathbb{R}}^{m\times e}$ encoded the same *e* = 3 experimental conditions as in the preliminary GLM analysis used for feature selection, described above. Following Equation (5), we pre-multiplied the data by the design to obtain the conditions by conditions matrix of parameters $Z^{-}Y$, which we sought to decompose into a weighted mixture of confounds $Z^{-}X$ and contrasts (hypotheses).

**Fig. 3:**
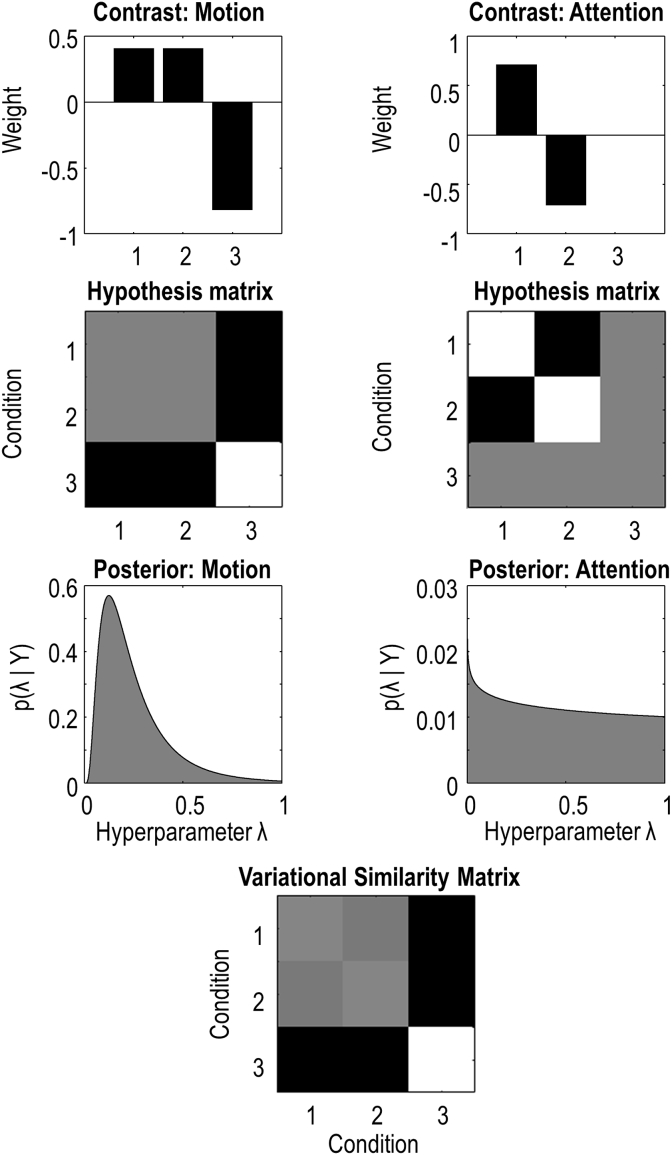
**Variational RSA of left V5 on the attention experiment. Top row**: Contrast vectors for each of the two contrasts, Motion and Attention. The three bars in each plot are the three experimental conditions: motion with attention, motion without attention and static dots. **Second row**: The same contrasts configured as matrices, where the three columns and rows correspond to the three experimental conditions. **Third row**: Posterior probability densities over the parameters quantifying the contribution of the motion contrast (left) and the attention contrast (right). **Bottom row**: The computed variational similarity matrix, i.e. the weighted contribution of motion and attention to the (second order) data. This corresponds to matrix **G** in Equation (7).

We defined two contrasts (Fig. 3, top row), which were the effects of *motion* and *attention* (vectors [1 1–2] and [1 -1 0], orthonormalized). Taking the outer product of each contrast with itself transposed $C_{i}=c_{i}c_{i}^{T}$ gave the corresponding hypothesis matrices (Fig. 3, second row). Each matrix *i* = 1 … 2 became a covariance component with a corresponding log scaling parameter $\lambda_{i}$. A further covariance component was included to model IID. errors. Finally, we estimated the hyperparameters using the Bayesian scheme described above.

### 4.2 Empirical results: left V5

Posterior estimates of the [hyper]parameters – quantifying the contribution of the two contrast matrices – are shown in Fig. 3 (third row). The motion parameter had a lognormal (*LN*) marginal posterior $p\left(\lambda_{1}|y\right)=LN\left(-2.09,0.49\right)$ and the attention parameter had marginal posterior $p\left(\lambda_{2}|y\right)=LN\left(-17.89,128\right)$. This means that there was a positive effect of motion – with expected value exp(−2.09)≈0.12, however there was little effect of attention, with expected value exp(−17.89)≈0.

We used Bayesian Model Reduction to compare this RSA model against reduced models where each parameter was fixed at its prior expectation; i.e., prior density $p\left(\lambda_{i}\right)=LN\left(-16,1/128\right)$. The posterior probability for the motion effect was 1.00 and for the attention effect the probability was 0.5, confirming the data were not sufficient to inform the presence or absence of attention. The resulting weighted mixture of the two components – the estimate of the similarity matrix **G** – is shown in Fig. 3 (bottom). The strong effect of motion and the very small effect of attention are readily visible, by comparison with the hypothesis matrices in the second row of the figure.

### 4.3 Searchlight analysis

A key application of RSA is to test the evidence for hypothesis matrices from different brain regions, modalities or even species. Here, we demonstrate this with variational RSA, by using the estimate of similarity matrix **G** from left V5 (Fig. 3, bottom row) as the hypothesis matrix for analysing all other brain regions. To do this, we moved a searchlight (sphere) through the grey matter of the whole brain and applied variational RSA with **G** as a single (multiple rank) component. In other words, we asked: where in the brain is there a set of voxels expressing the same mixture of experimental effects as observed in left V5?

**Fig. 4:**
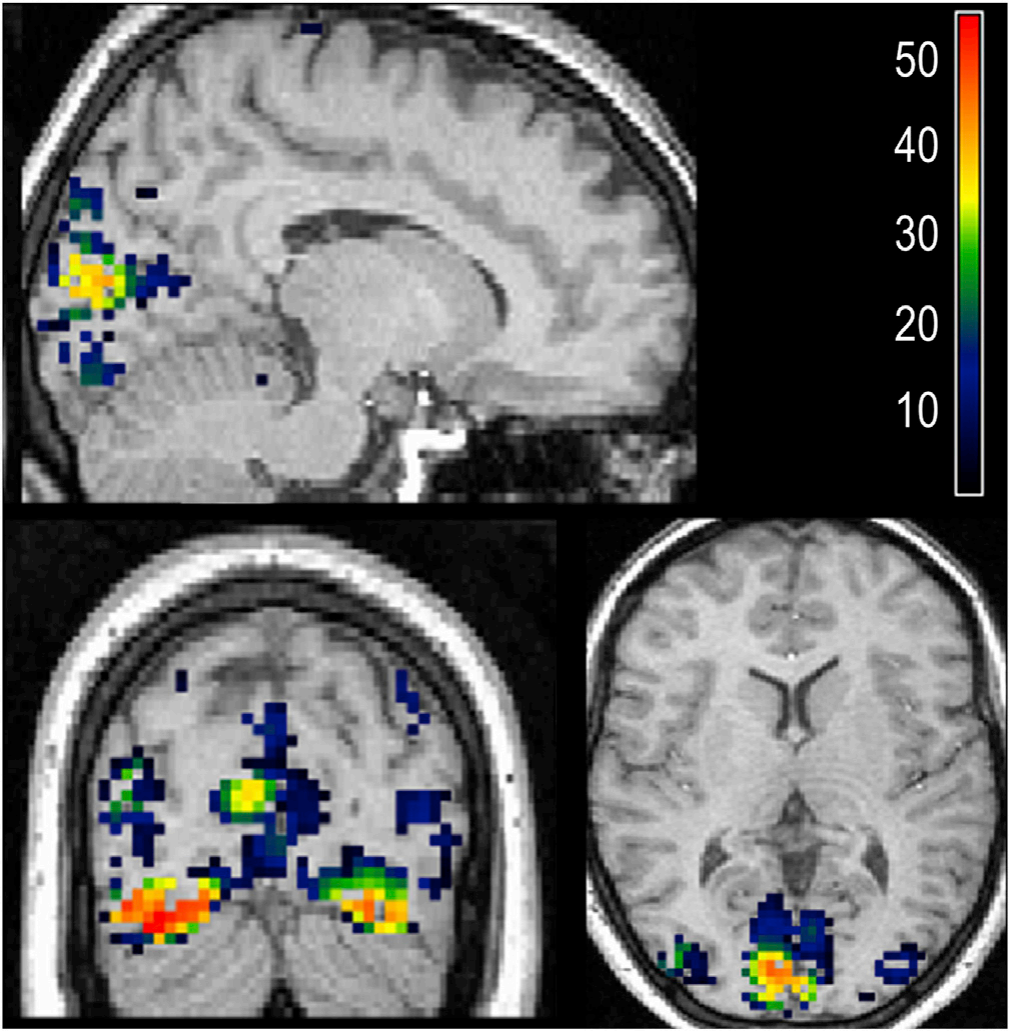
**Searchlight analysis over all grey matter voxels.** The coloured voxels indicate the Log Bayes factor for each RSA model with a hypothesis matrix derived from left V5, relative to a null model with this component fixed to nearly zero. This log evidence map is thresholded at a posterior probability greater than 0.95. Slices are positioned at MNI coordinates x = −13, y = −82 and z = 10, where left is shown on the left, projected onto slices from the subject's structural MRI.

To assess this quantitatively, we compared the evidence for each searchlight's RSA model against a null model, in which the V5 component was suppressed by setting the prior: p(λ)=LN(−16,1/128). This amounts to testing for a non-trivial expression of V5-like response profiles. Fig. 4 shows the ensuing *log evidence map* (i.e., map of log Bayes factors) in favour of the full model, thresholded at a posterior probability of 95%. As expected, the strongest effects were seen in bilateral V5, as well as in primary visual cortex around the calcarine sulcus.

## 5 Discussion

In summary, multivariate analysis of neuroimaging data (RSA and PCM) can be treated as a covariance component estimation problem, where each hypothesis is encoded as a covariance component and the contribution of the components are estimated using standard variational Bayesian methods. Here, we have illustrated two ways of specifying the hypotheses. Either one can test for distributed responses in terms of a single profile (contrast vector) over experimental conditions or stimulus features, using *t*-contrasts in classical multivariate analyses. This takes the form of a rank one hypothesis matrix in covariance component analyses. Alternatively, one can test for the presence of a mixture of profiles using *F*-contrasts—or hypothesis matrices with a rank greater than one in covariance component analysis. The key difference between classical multivariate analyses, (e.g., MANOVA, canonical correlation analysis, *etc*) and covariance component analyses (e.g., PCM, representational similarity analysis *etc*) boils down to a parameterisation of distributed responses in terms of first-order responses, **U**, versus second-order effects, **G** = **UU**^*T*^. So, which is the most appropriate?

The answer depends upon whether one is interested in the pattern of responses over voxels in spatial imaging (or time in timeseries analysis). The advantage of characterising responses in terms of first-order parameters is that one can estimate the spatial (or temporal) pattern of distributed responses. However, if this pattern is not of interest (or is not conserved over subjects) then testing for covariance components could be more appropriate; especially if one wants to make inferences at the between-subject level. This paper effectively describes the requisite random effects modelling of second-order parameters (i.e., hyperparameters) using parametric empirical Bayes.

The distinction between first and second-order parameterisation is prescient in the analysis of electrophysiological timeseries. In this setting, one has to choose between the analysis of evoked (first-order) responses as a function of peristimulus time and induced (second-order) responses, usually as a function of frequency. The key difference rests upon whether one believes that systematic responses are conserved over peristimulus time; namely, that the temporal profile or shape of an evoked response matters. Conversely, if the temporal profile of responses is not conserved over trials or subjects, then the power of induced responses is the more appropriate characterisation. Indeed, as noted in the introduction, the procedures described in this paper are used routinely along these lines in electromagnetic source reconstruction (Friston et al., 2008b). The analogy for spatial imaging (e.g., fMRI) is that the spatial pattern induced by a particular stimulus attribute over voxels is not in itself interesting and, more importantly, not conserved over subjects. In this context, component analysis would be more appropriate.

Finally, the foregoing discussion speaks to a key choice when using component analyses. This is the choice between decomposing any given hypothesis matrix into its orthogonal patterns or retaining the particular mixture of patterns when defining a component of interest. This choice equips RSA with the latitude to test each orthogonal constituent of a covariance component or ‘lock in’ a specified mixture of induced patterns as a single covariance component—a component that characterises the functional specialisation of a brain region. The former is most useful if a region could represent multiple components, but the relative weightings of those components are unknown in advance. Whereas, the latter is useful if one wishes to test a specific hypothesis about whether a combination of components with particular weightings is present in the observed pattern of responses.

## 6 Conclusion

In conclusion, this technical note describes a standard (variational) implementation of covariance component analysis that has all the functionality of pattern component modelling and representational similarity analysis. It does not rely upon sampling or cross validation and is therefore efficient (in the sense of the Neyman Pearson lemma). It deals gracefully with spatial correlations and allows the user to specify (hyper) priors over competing pattern or component hypotheses. It allows one to specify rank one hypothesis matrices (as in standard hypothesis testing of main effects and interactions) or full rank hypotheses (as empirical covariance components from other brain regions, sessions, subjects or species). The applications we have in mind in the latter case would enable people to answer questions of the following kind: does the mixture of condition-specific responses found in V1 provide a good account of responses in the frontal eye fields—or do I need to consider other mixtures, say from the orbital prefrontal cortex? We hope that questions like this can be posed to data efficiently using the scheme above.

## Software note

The procedures described in this note can be accessed from the results panel of the (next release of) SPM GUI (labelled RSA). The key routines that implement the analyses reported in the figures of this paper are **spm_reml_sc.m**, **spm_dcm_peb.m** and **spm_log_evidence.m**. These routines are available as Matlab code in the SPM academic software: http://www.fil.ion.ucl.ac.uk/spm/. The simulations in this paper can be reproduced (and customised) via a graphical user interface by typing ≫ DEM and selecting the **CVA & RSA** demo.

## Disclosure statement

The authors have no disclosures or conflict of interest.

## Footnotes

1 For simplicity, we are restricting ourselves to dissimilarity matrices based upon mean centred, Euclidean normalised data.

2 Note that the confounds – and associated parameters – do not appear explicitly in Equation (5) because we are, effectively, working in the null space of the confounds (i.e., the confounds are absorbed into the residual forming matrix).

3 By *ad hoc*, we mean that a posterior over a random variable is replaced with point estimator.

4 This does not mean to say that variational RSA is magically immune to high levels of observation noise or low degrees of freedom in the data. This is because the uncertainty about the hyperparameters (and ensuing inference) is influenced by degrees of freedom and noise levels; especially when there are conditional dependencies between the hyperparameter estimates.
